# Evaluation of DMSO-free cryopreservation reagent XT-Thrive for establishment of mesenchymal stem cell bank platform

**DOI:** 10.3389/fbioe.2026.1736526

**Published:** 2026-02-05

**Authors:** Alan Tin-Lun Lam, Arthi Shridhar, Harish K. Handral, Jialing Lee, Steve Oh, Xiaoxi Wei

**Affiliations:** 1 Bioprocessing Technology Institute (BTI), Agency for Science, Technology and Research, Singapore, Singapore; 2 X-Therma Inc., Hercules, CA, United States; 3 Singapore Institute of Food and Biotechnology Innovation (SIFBI), Agency for Science, Technology and Research, Singapore, Singapore; 4 Vin University, Hanoi, Vietnam

**Keywords:** biobanking, cryopreservation, DMSO-free, manufacturing, mesenchymal stem cells

## Abstract

**Introduction:**

Effective cryopreservation is essential for the clinical application and large-scale banking of mesenchymal stem cells (MSCs). This study compares the performance of a novel DMSO-free cryoprotectant, XT-Thrive®, with a conventional DMSO-based solution, CryoStor® CS10, in preserving both commercial and donor-derived bone marrow MSCs (BM-MSCs). Evaluations focused on viability, recovery, proliferation, and functional characteristics across master cell bank (MCB), working cell bank (WCB), and final product (FP) stages.

**Methods:**

In Part 1, commercial BM-MSCs were cryopreserved in XT-Thrive or CS10 and evaluated for pre-freeze viability, post-thaw survival (up to 6 h), and recovery in 2D and 3D cultures. In Part 2, donor-derived BM-MSCs were cryopreserved at passages 2 (MCB), 4 (WCB), and 8 (FP), and assessed for cumulative population doubling levels (cPDL), immunophenotype, clonogenicity, differentiation potential, secretome profile, telomere length, karyotype stability, and tumorigenicity.

**Results:**

XT-Thrive–preserved MSCs maintained >90% pre-freeze viability after 24-h room temperature holding, compared to a ∼40% drop with CS10. Post-thaw viability at 6 h remained above 85% with XT-Thrive, vs. 60%–70% with CS10. In 3D microcarrier cultures under serum-free conditions, XT-Thrive-preserved MSCs demonstrated a ∼2.5-fold improvement in viable cell recovery compared to CS10, which failed to support recovery and expansion. XT-Thrive–preserved donor MSCs showed significantly higher cPDL at passages 8 FP (19.8 ± 0.4 vs. 15.4 ± 0.5, *p* < 0.001). CFU-F efficiency was also higher (∼23% vs. ∼15%). Furthermore, XT-Thrive–preserved MSCs exhibited enhanced osteogenic differentiation and increased secretion of FGF2 and HGF (1.8-fold and 2.1-fold increase, respectively), without compromising karyotype integrity, telomere length, or safety *in vivo*.

**Conclusion:**

XT-Thrive provides superior pre-freeze stability, post-thaw recovery, expansion potential, and osteogenic functionality compared to CS10, while maintaining MSC identity and genomic stability. These results support XT-Thrive as a promising DMSO-free alternative for clinical-grade MSC biobanking and manufacturing.

## 1 Introduction

Mesenchymal stem cells (MSCs) are multipotent stromal cells found in various tissues such as bone marrow, adipose tissue, and umbilical cord. They can differentiate into osteogenic, chondrogenic, and adipogenic lineages, expand robustly *ex vivo*, and exert therapeutic effects mainly through paracrine signaling (Zhou and Shi, 2023). These secreted factors promote angiogenesis, modulate immune responses, and support tissue regeneration. Their ease of isolation, scalability, and low immunogenicity underpin their widespread investigation as therapies for inflammatory, degenerative, and autoimmune diseases.

From a manufacturing perspective, MSCs are among the most clinically advanced cell therapy products. Their scalability, compatibility with cryopreservation, and well-established protocols for culture and quality facilitate translation into regulatory compliance. To date, over 1,000 clinical trials have explored MSC therapies, and the first regulatory approval for graft-versus-host disease (GVHD) highlights their therapeutic promise (Blanc et al., 2025).

Cryopreservation is central to MSC manufacturing, enabling master cell banks (MCBs) and working cell banks (WCBs), extended storage, and final production formulation. Post-thaw viability and function are essential for potency safety (Brezinger-Dayan et al., 2022; Bahsoun et al., 2020; Oja et al., 2019). In workflows involving direct infusion or minimal manipulation, where cells are administrated immediately after thaw, cryopreservation is critical. In high-cost therapies such as CAR-T, where per-dose manufacturing exceed USD 50,000, robust cryopreservation protocols are critical to ensuring timely delivery of viable cells (Hernandez et al., 2018).

Dimethyl sulfoxide (DMSO) remains the standard cryoprotectant but raises significant safety and regulatory concerns. Even at low concentrations (0.5%–1.5%), DMSO is linked to toxicities ranging from nausea to arrhythmias and may disrupt nucleic acids, proteins, and induce epigenetic changes (Weng and Beauchesne, 2020; Galvao et al., 2014). Furthermore, studies have shown that while DMSO supports cell survival, it can also induce apoptosis, oxidative stress, and DNA damage (Erol et al., 2021; Ding et al., 2024). Clinically, MSCs are typically cryopreserved in media containing 5%–10% DMSO. Efforts to reduce rick–such as increasing cell concentration or post-thaw washing–introduce logistical challenges and fail to fully mitigate DMSO’s toxicity (Ginis et al., 2012; Chen et al., 2013).

In this study, we evaluate XT-Thrive®, a novel, chemically defined, peptide-based, DMSO-free cryopreservation reagent developed by X-Therma Inc. While XT-Thrive has demonstrated superior preservation of hematopoietic stem cells (HSCs) and peripheral blood mononuclear cells (PBMCs) (Gilfanova et al., 2021), yet its application in MSC banking remains underexplored. Here, we compare XT-Thrive with CryoStor® CS10, a widely used DMSO-based reagent to preserve both commercial and donor-derived BM-MSCs across multiple manufacturing-relevant conditions, including 2D and 3D culture, pre-freeze and post-thaw holding. We further assess the impact on phenotypic and functional characteristics, including proliferation, differentiation, secretory activity, genomic stability, and tumorigenicity.

Our findings highlight the potential of XT-Thrive to address key limitations of DMSO-based reagents, supporting its use as a safer and more flexible solution for scalable, clinically compliant MSC biobanking.

## 2 Materials and methods

### 2.1 Human BM-MSC culture

This study comprised two parts: (1) commercially available BM-MSCs and (2) primary BM-MSCs freshly isolated from donors.

Part 1: Pre-frozen BM-MSCs (passage 2; Lonza PT-2501were expanded in T-175 flasks (5,000 cells/cm^2^) using a 10% fetal bovine serum (FBS)-DMEM until ∼80% confluency. Cells were then maintained in either serum-containing medium (SCM; 10% FBS-DMEM) or serum-free medium (SFM; 5% human platelet lysate [hPL]-DMEM). After reaching ∼80% confluency, cells were cryopreserved using either CryoStor® CS10 (Biolife Solutions) or XT-Thrive® (X-Therma Inc.) as detailed in Section 2.2.

Following 1 week of liquid nitrogen (LN_2_) storage, cells were thawed (Section 2.3) and further expanded for 6 days in either SCM or SFM, either as monolayers (T-75 flasks) or on PlasticPlus™ microcarriers (SoloHill) in 125-mL Erlenmeyer shake flasks (Corning) seeded at 5,000 cells/cm^2^, as previously described (Lam et al., 2019).

Part 2: Donor-derived BM-MSCs were isolated from bone marrow aspirates (healthy donors, 21–60 years) with informed consent and Institutional Review Board approval (NHG DSRB, Singapore; Ref: 2019/00284). Mononuclear cells were obtained by density gradient centrifugation (Lymphoprep™, StemCell Technologies) (Ling et al., 2020), plated at 40–50 ×10^6^ cells per T-175 flask, and expanded in SFM (5% hPL-DMEM). Cultures were passaged to generate a master cell bank (MCB; passage 2), working cell bank (WCB; passage 4), and final product (FP; passage 8) (Lechanteur et al., 2016). All cultures in Part 2 were maintained in 2D T-175 flasks at 5,000 cells/cm^2^ in SFM.

### 2.2 Cryopreservation

In both parts of the study, BM-MSCs were resuspended at 1 ×10^6^ cells/mL in XT-Thrive or CS10, aliquoted into 1-mL cryovials (n = 3 per group), frozen in a Mr. Frosty™ container (ThermoFisher Scientific), and stored in LN_2_. Cells were thawed with the ThawSTAR® automated thawing system (Biolife Solutions) unless otherwise specified for holding studies (Section 2.3).

### 2.3 Pre-freeze and post-thaw holding studies

To evaluate robustness, cell viability was assessed after prolonged pre-freeze and post-thaw exposure, but without wash steps.

Pre-freeze holding: Cells were incubated with XT-Thrive or CS10 for 0, 4, or 24 h at room temperature prior to freezing.

Post-thaw holding: Thawed cryovials were held at room temperature for 0, 2, 4, and 6 h (Part 1) or 0 and 4 h (Part 2).

Viability and cell counts were assessed using the NucleoCounter® NC-3000™ (Chemometec) and Acridine Orange/DAPI (AO/PI) staining.

### 2.4 Post-thaw cell recovery and growth

In Part 1, thawed cells were cultured for 6 days in SCM or SFM, as monolayers (T-75 flasks) or microcarrier suspension (40 rpm, 125-mL flasks) In Part 2, post-thaw recovery was assessed at MCB (P2), WCB (P4), and FP (P8) under monolayer SFM conditions, to simulate a typical needle to needle manufacturing process.

Monolayer cultures: Total viable cells were quantified using AO/PI staining.

Microcarrier cultures: 200 µL samples were stained with DAPI for total viable cell counts; 500 µL samples were stained with AO/DAPI for live imaging (EVOS™ M5000 microscope).

Population doubling level (PDL) was calculated using the formula:

$$PDL=\left[\mathrm{Log}\left(N_{x}\right)-\mathrm{Log}\left(N_{0}\right)\right]÷\mathrm{Log}\left(2\right)$$

Where N_0_ is the seeding density and N_x_ is the final cell count. Cumulative PDL (cPDL) was calculated by summing PDL across passages (Srinivasan et al., 2022).

### 2.5 Immunophenotyping

MSC identity was confirmed by flow cytometry using ISCT criteria. Cells were stained with fluorophore-conjugated antibodies against CD73, CD90, CD105 (positive) and CD34, CD45 (negative) (BioLegend, 1:100 dilution). Cells were analyzed on a NovoCyte™ 3,000 flow cytometer (Agilent) after washing and resuspension in 1% BSA/PBS.

### 2.6 CFU-F assay

Colony-forming unit-fibroblast (CFU-F) efficiency was determined by seeding 500 cells/60-mm dish (n = 3/condition) in α-MEM+10% FBS. After 14 days, colonies were fixed, stained with 0.5% crystal violet and counted. CFU-F efficiency was calculated as:

$$\text{CFU}-F=\frac{Number\;of\;Colonies\;counted}{Number\;of\;Seeded\;Cells}\times 100\%$$

### 2.7 Trilineage differentiation

Differentiation into adipogenic, osteogenic, and chondrogenic lineages was performed using STEMPRO® hMSC differentiation kits (ThermoFisher) as per manufacturer protocols. Differentiation was visualized using Oil Red O (adipogenesis), Alizarin Red (osteogenesis), and Alcian Blue (chondrogenesis) staining (Lam et al., 2023).

### 2.8 Cytokine profiling

Conditioned media were analyzed using a Bio-Plex® Multiplex Assay (Bio-Rad). Cytokines were quantified against recombinant protein standards in 5% hPL-DMEM. Detection was performed with biotinylated antibodies and streptavidin-PE on Bio-Plex system (Lam et al., 2019).

### 2.9 Telomere length measurement

Absolute telomere length (aTL) was quantified in P8 cells using a qPCR-based assay as previously described (Samsonraj et al., 2013).

### 2.10 Karyotyping

Cytogenetic analysis of P8 BM-MSCs was performed by the Cytogenetics Laboratory, Singapore General Hospital, to detect any chromosomal abnormalities (Ling et al., 2020).

### 2.11 Tumorigenicity assay

NOD-SCID mice were injected subcutaneously with 2 ×10^6^ BM-MSCs (XT-Thrive- or CS10-preserved, P8). Mice were monitored for 12 weeks before histological evaluation for tumor formation or toxicity.

### 2.12 Statistical analysis

Data are presented as mean ± standard deviation (SD). Analyses were conducted in GraphPad Prism (v10.3.1) using one-way or two-way ANOVA, with appropriate post hoc. A p-value <0.05 was considered statistically significant.

## 3 Results

### 3.1 Part 1: comparative evaluation of XT-Thrive and CS10 in cryopreserving commercial BM-MSCs

#### 3.1.1 Cell viability following pre-freeze and post-thaw holding

Commercial BM-MSCs expanded in either serum-containing medium (SCM; 10% FBS-DMEM) or serum-free medium (SFM; 5% hPL-DMEM) were incubated with XT-Thrive or CS10 at room temperature for 2, 4, or 24 h prior to cryopreservation.

**FIGURE 1:**
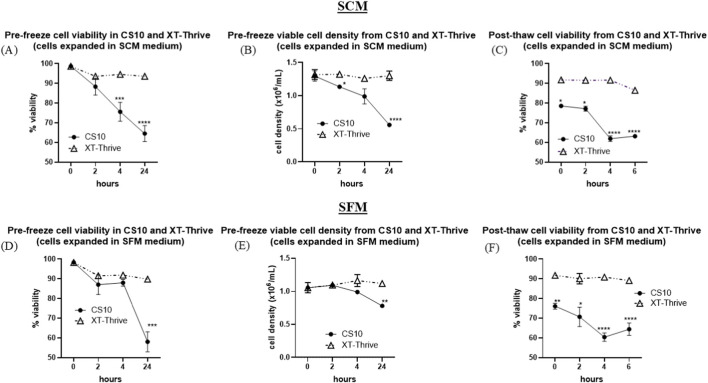
Pre-freeze and post-thaw viability of BM-MSCs cryopreserved in XT-Thrive vs. CS10. **(A,B)** Cell viability and total cell number after 0, 2, 4, and 24-h pre-freeze incubation with cryoprotectant in serum-containing medium (SCM). **(C)** Post-thaw viability at 0, 2, 4, and 6 h in SCM. **(D,E)** Pre-freeze incubation in serum-free medium (SFM). **(F)** Post-thaw viability in SFM.

As shown in Figure 1, XT-Thrive preserved high cell viability and total cell numbers up to 24 h in both SCM-derived (Figures 1A,B) and SFM-derived cells (Figures 1D,E), with no significant decline observed. In contrast, CS10-exposed cells showed reduced viability within the first 2 hours—approximately 10% loss in both SCM (Figures 1A,B) and SFM groups (Figures 1D,E)—and a substantial decline (∼33% and ∼40%, respectively) after 24 h (p < 0.0001). These findings suggest that XT-Thrive confers significantly greater stability during prolonged pre-freeze handling.

Post-thaw viability was then assessed at 0, 2, 4, and 6 h after thawing. XT-Thrive–preserved cells retained ∼90% viability up to 6 h post-thaw in both SCM (Figure 1C)- and SFM-expanded groups (Figure 1F). In contrast, CS10-preserved cells exhibited a ∼20% immediate viability drop and a further decline of ∼40% by 6 h (p < 0.0001; Figures 1C,F). These results highlight the superior post-thaw stability of XT-Thrive–preserved MSCs under extended handling conditions.

#### 3.1.2 Post-thaw recovery and growth performance

Thawed BM-MSCs were seeded without washing into both monolayer and microcarrier cultures, in SCM and SFM media.

**FIGURE 2:**
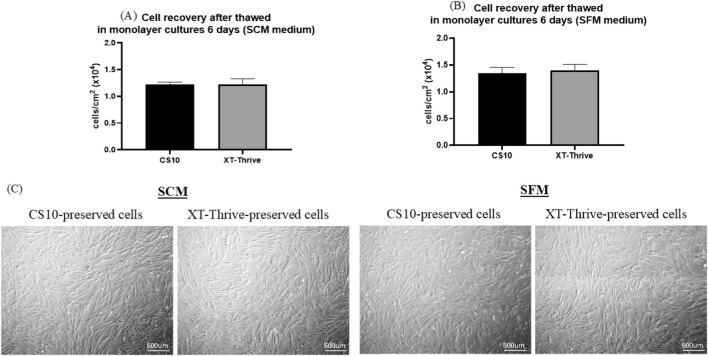
Post-thaw growth recovery in monolayer cultures. **(A,B)** Viable cell recovery after 6 days in SCM and SFM, comparing XT-Thrive and CS10. **(C)** Representative cell morphology images showing no observable differences between cryoprotectants in both media conditions.

In monolayer cultures, after 6 days, total viable cell counts were comparable between XT-Thrive and CS10 across conditions, as shown in Figures 2A,B. Cell morphology remained spindle-shaped and consistent across all groups (Figure 2C).

**FIGURE 3:**
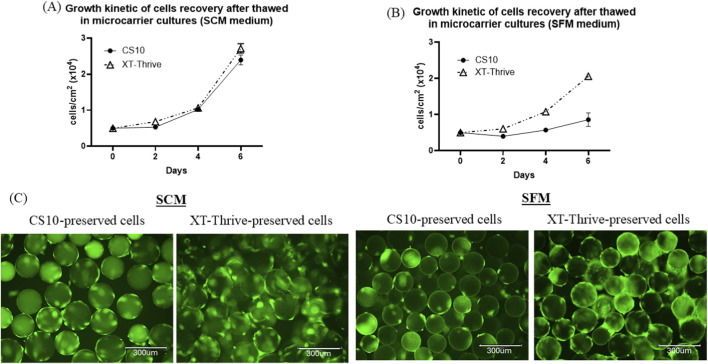
Post-thaw growth recovery in microcarrier cultures. Growth of XT-Thrive–preserved and CS10-preserved MSCs over 6 days under **(A)** SCM and **(B)** SFM conditions. **(C)** Fluorescence imaging (AO/DAPI) showing higher viability and cell density with XT-Thrive on PlasticPlus™ microcarriers.

In microcarrier cultures, XT-Thrive-preserved exhibited comparable expansion compared to CS10, in SCM conditions (Figure 3A). In contract, XT-Thrive–preserved cells demonstrated markedly superior expansion, ∼2.5-fold higher viable cell recovery than CS10 (Figure 3B), in SFM conditions. Fluorescence imaging confirmed a visible higher cell attachment and viability on microcarriers in XT-Thrive groups especially in the SFM condition (Figure 3C).

Flow cytometric analysis confirmed consistent MSC marker expression (CD73^+^, CD90^+^, CD105^+^, CD34^−^, CD45^−^) across all conditions, indicating preservation of MSC identity regardless of cryoprotectant, medium, or culture platform (Supplementary Figure S1A,S1B).

Colony-forming efficiency was also comparable between groups, with no statistically significant difference in CFU-F counts (Supplementary Figure S2A,S2B), supporting maintenance of clonogenic potential following cryopreservation.

### 3.2 Part 2: evaluation of XT-Thrive and CS10 for donor-derived primary BM-MSC biobanking

#### 3.2.1 Cell viability at different banking stages

Donor-derived BM-MSCs were evaluated at three stages of the manufacturing workflow: MCB (P2), WCB (P4), and FP (P8).

**FIGURE 4:**
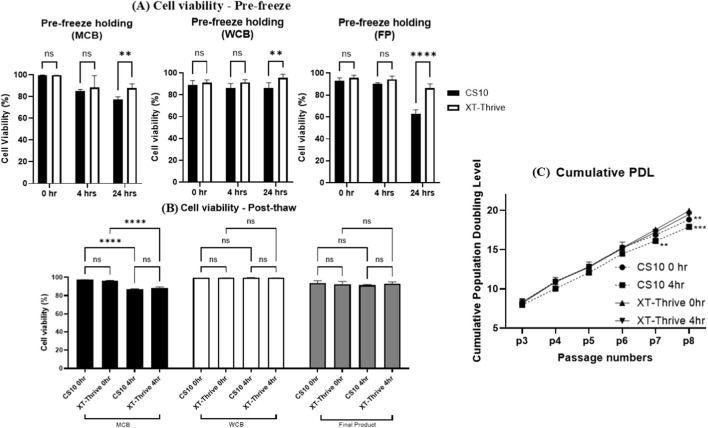
Cryopreservation performance in donor-derived BM-MSCs across cell banking stages. **(A)** Pre-freeze viability after 24-h room temperature incubation at MCB, WCB, and FP stages. **(B)** Post-thaw viability after 4-h room temperature holding. **(C)** Cumulative population doubling level (cPDL) from P3 to P8.

As shown in Figure 4A - after 24-h pre-freeze holding at room temperature, XT-Thrive–preserved cells showed higher viability than CS10. This difference was particularly significant at the FP stage (∼80% vs. ∼60%, p < 0.0001; Figure 4A), suggesting enhanced tolerance to delayed cryopreservation with XT-Thrive.

Post-thaw viability was assessed after a 4-h hold at room temperature, simulating the clinical thaw-to-infusion window, as shown in Figure 4B. At the WCB and FP stages, both XT-Thrive and CS10 preserved viability effectively, with no significant differences between groups. However, at the MCB stage, viability decreased significantly in both conditions after 4 h (Figure 4B, p < 0.0001), indicating increased sensitivity of early-passage MSCs to extended post-thaw handling.

#### 3.2.2 Proliferative recovery after thaw

The proliferative capacity of thawed MSCs was evaluated by cumulative population doubling level (cPDL). XT-Thrive–preserved cells consistently demonstrated higher cPDL values compared to CS10, with significant differences observed at passages 7 and 8 (p < 0.01 and p < 0.001, respectively; Figure 4C). Cell morphology however, remained normal across all conditions (Supplementary Figure S3), suggesting that improved expansion did not compromise phenotypic integrity.

#### 3.2.3 Final product characterization at passage 8

Immunophenotyping: BM-MSCs retained the typical ISCT-defined marker expression at passage 8, with no significant differences between XT-Thrive and CS10 regardless of holding time (Supplementary Figure S4A).

Colony-Forming Efficiency: XT-Thrive–preserved cells exhibited higher CFU-F potential compared to CS10, independent of holding duration (Supplementary Figure S4B), suggesting potentially greater clonogenic capacity.

**FIGURE 5:**
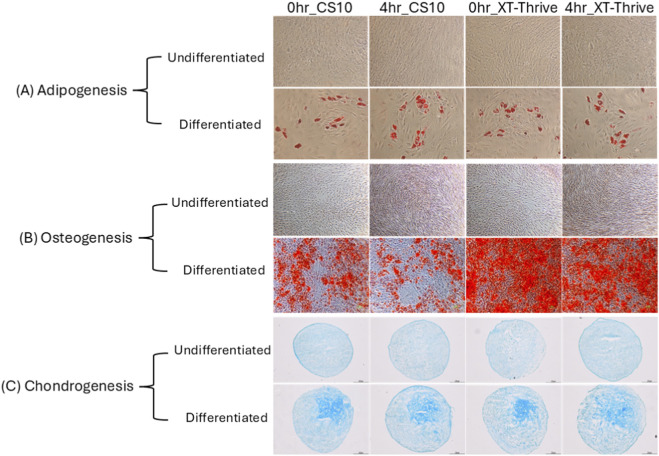
Trilineage differentiation of cryopreserved BM-MSCs at FP stage (passage 8). **(A)** Adipogenic differentiation (Oil Red O staining). **(B)** Osteogenic differentiation (Alizarin Red staining). **(C)** Chondrogenic differentiation (Alcian Blue staining). XT-Thrive–preserved cells showed enhanced osteogenic differentiation.

Trilineage Differentiation: Both groups retained adipogenic, osteogenic, and chondrogenic potential as evidenced by Oil Red O, Alizarin Red, and Alcian Blue staining, respectively (Figures 5A–C). Notably, XT-Thrive–preserved cells demonstrated more robust osteogenic differentiation, as shown by increased staining intensity (Figure 5B).

**FIGURE 6:**
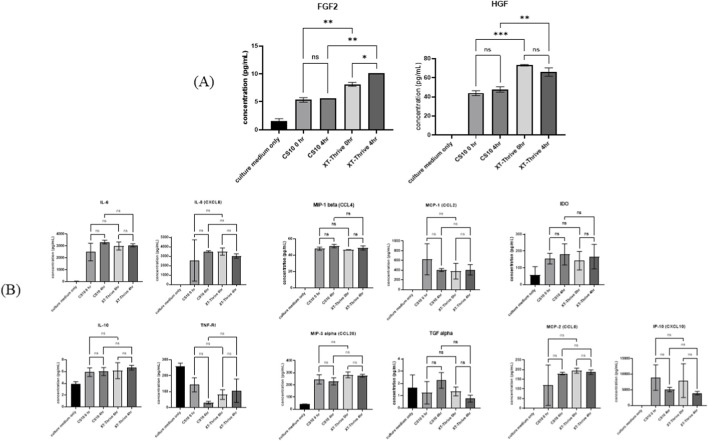
Cytokine secretion profile of thawed BM‐MSCs at FP stage (passage 8). **(A)** XT‐Thrive–preserved cells showed significantly elevated levels of FGF2 and HGF. **(B)** Other cytokines showed no major differences compared to CS10.

Secretome Analysis: Of 21 cytokines evaluated, 13 were detected. XT-Thrive–preserved cells secreted higher levels of FGF2 and HGF compared to CS10-preserved cells (Figure 6A). These factors are known to promote osteogenesis and may explain the enhanced differentiation observed. There were no significant differences for the other cytokines measured (Figure 6B).

Telomere Length: Quantitative PCR analysis showed no significant differences in absolute telomere length (aTL) between groups at passage 8, indicating similar replicative histories (Supplementary Figure S5).

Karyotype Integrity: Cytogenetic analysis confirmed the absence of chromosomal abnormalities in both XT-Thrive–and CS10-preserved BM-MSCs at passage 8 (Supplementary Figure S6).

Tumorigenicity: No macroscopic tumor formation (Supplementary Figure S7) or histopathological abnormalities (data not shown) were observed in NOD-SCID mice injected with preserved BM-MSCs from either group after 12 weeks, supporting the safety of both cryopreservation methods.

Summary: Across both parts of the study, XT-Thrive demonstrated superior pre-freeze and post-thaw stability, improved proliferative capacity, and enhanced osteogenic and adipogenic differentiation, while maintaining safety, genomic stability, and key MSC characteristics. These findings highlight XT-Thrive as a promising DMSO-free alternative for clinical-grade MSC biobanking.

## 4 Discussion

XT-Thrive, a novel DMSO-free and GMP-grade cryopreservation reagent, has previously demonstrated superior preservation outcomes for HSCs and PBMCs (Gilfanova et al., 2021). Building on these findings, we sought to evaluate its applicability for MSC biobanking. In this study, we systematically compared XT-Thrive with the conventional DMSO-based CryoStor CS10, assessing their effects on pre-freeze/post-thaw cell viability, recovery, expansion potential, and functional attributes, spanning both commercial and donor-derived BM-MSCs across key stages of the cell manufacturing workflow.

In Part 1, commercially sourced BM-MSCs were used to establish baseline comparisons. Notably, XT-Thrive demonstrated superior performance during the 24-h pre-freeze holding period at room temperature, maintaining viability above 90%, in contrast to CS10, which showed a marked decline to ∼60% (Figures 1A,D). Maintaining membrane integrity and metabolic stability during this phase is essential, as compromised pre-freeze viability can impair post-thaw functionality and reduce recovery efficiency (Oja et al., 2019; Pal et al., 2008; Cottle et al., 2022). The stability offered by XT-Thrive may thus confer a significant advantage in large-scale manufacturing scenarios where processing delays or logistics-related holding periods are common (Chen et al., 2013).

Post-thaw viability also favored XT-Thrive, with cells maintaining >85% viability even after 6 h at room temperature (Figures 1C,F). This resilience is particularly valuable in clinical settings, where the thaw-to-infusion window may extend beyond the typical 2–4 h. Interestingly, XT-Thrive–preserved MSCs exhibited enhanced proliferation in 3D microcarrier suspension cultures—an observation not seen in CS10-preserved cells under SFM conditions (Figure 3). The improved attachment and expansion could reflect better preservation of cytoskeletal and membrane-associated signaling pathways, which are especially critical in dynamic culture systems (Whaley et al., 2021; Bahsoun et al., 2020). Given the increasing shift toward bioreactor-based manufacturing, the compatibility of XT-Thrive with microcarrier systems enhances its translational value.

Both reagents preserved core MSC properties, including immunophenotype and CFU-F capacity (Supplementary Figures S1,S2), indicating that neither compromised basic MSC identity. Taken together, the results from Part 1 support the use of XT-Thrive as a process-compatible reagent suited for scalable and GMP-compliant MSC biobanking workflows.

In Part 2, we extended the evaluation to donor-derived primary BM-MSCs and examined reagent performance across the cell banking continuum—from MCB (P2) to WCB (P4) and final product (P8). XT-Thrive preserved higher cell viability following 24-h pre-freeze holding, particularly at the FP stage (∼80% vs. ∼60% for CS10; Figure 4A), confirming its robustness under prolonged handling conditions. These findings are consistent with known cytotoxic effects of DMSO, which can disrupt membrane function, induce oxidative damage, and alter protein stability (Weng and Beauchesne, 2020).

Post-thaw viability at 4 h remained high in both groups at WCB and FP stages. However, a significant decline was observed at the MCB stage, regardless of reagent (Figure 4B), suggesting heightened sensitivity of early-passage MSCs to post-thaw stress. This may reflect incomplete adaptation to *in vitro* conditions or heightened oxidative susceptibility. Further studies investigating mitochondrial integrity, ROS production, or apoptosis markers in early-passage cells would help elucidate the underlying mechanisms.

XT-Thrive–preserved MSCs also demonstrated significantly higher cumulative population doubling levels at later passages (Figure 4C), suggesting enhanced proliferative potential. Increased cPDL can improve yield, reduce the need for additional donor sourcing, and extend the functional lifespan of cell banks. Notably, CFU-F efficiency was higher in XT-Thrive-preserved cells compared to CS10, regardless of post-thaw holding (Supplementary Figure S4B), further support superior clonogenic preservation. However, it is well-established that excessive passaging (typically beyond cPDL ∼40) can promote senescence and genetic instability (Oliver-Vila et al., 2016; Yang, 2018; Yang et al., 2018). In our study, XT-Thrive–preserved cells reached ∼20 cPDL by passage 8, which lies within the optimal range for clinical application (Lechanteur et al., 2016; Pakzad et al., 2022).

Comprehensive characterization at the final product stage further validated XT-Thrive’s suitability for clinical translation. Immunophenotype, telomere length, and karyotype integrity were preserved across both reagents (Supplementary Figures S4A, S5, S6). Both groups retained trilineage differentiation potential; Surprisingly, XT-Thrive–preserved MSCs demonstrated enhanced osteogenic differentiation, with stronger Alizarin Red staining (Figure 5B).

Notably, cytokine profiling revealed increased secretion of FGF2 and HGF in XT-Thrive–preserved cells (Figure 6), both of which are known to enhance osteogenesis and support angiogenesis (Aenlle et al., 2014; Byun et al., 2014; Grano et al., 1996; Ito et al., 2008; Park et al., 2022). This may partially explain the observed functional advantage and suggest that XT-Thrive not only preserves basic MSC characteristics but may also better maintain or even enhance key regenerative properties.

*In vivo* tumorigenicity studies confirmed the safety of both cryopreservation approaches, with no evidence of tumor formation or systemic toxicity following subcutaneous implantation (Supplementary Figure S7). This is a critical consideration for regulatory approval and supports the translational readiness of XT-Thrive as a cryopreservation reagent.

Overall, XT-Thrive presents multiple advantages over CS10: (1) improved pre-freeze and post-thaw stability, (2) better compatibility with microcarrier and serum-free systems, (3) enhanced proliferative and differentiation capacity, and (4) preserved genomic integrity and safety. These findings support its adoption in clinical-grade MSC biobanking workflows, particularly for advanced manufacturing environments requiring flexible and non-toxic preservation solutions.

Recent multicenter studies have established important benchmarks for MSC cryopreservation, demonstrating that acceptable post-thaw viability and phenotypic identity can be achieved across sites using standardized, DMSO-containing formulations (Mamo et al., 2024). However, these studies also highlight persistent translational limitations, including reliance on DMSO, variable post-thaw functional recovery, and limited assessment of therapeutic-relevant cell performance.

In contrast, our present study demonstrates that XT-Thrive confers multiple advantages that extend beyond basic post-thaw viability, several of which were directly evaluated in our experimental system. XT-Thrive-preserved MSCs not only exhibited improved post-thaw recovery, including enhanced attachment efficiency, metabolic activity, and proliferation kinetics, but evaluated immunomodulatory effects, parameters are critical for clinical efficacy yet are not routinely examined in other cryopreservation studies. A key differentiator of XT-Thrive is that it is a single component, DMSO-free formulation. Continued dependence on DMSO-based cryoprotectants presents safety and manufacturing challenges, including infusion-related toxicities and the need for post-thaw washing steps that can reduce cell yield and introduce variability. XT-Thrive eliminates these constraints while maintaining or improving post-thaw MSC performance, thereby offering a safer and more streamlined workflow compatible with off-the-shelf and point-of-care cell therapy applications (Supplementary Table S1).

Together, these findings position XT-Thrive as a next-generation cryopreservation solution that not only meets established benchmarks but also advances the field by preserving functional potency and recovery capacity in a DMSO-free format.

## 5 Conclusion

We evaluated XT-Thrive®, a chemically defined, DMSO-free cryopreservation reagent, against the conventional DMSO-based CryoStor® CS10 for mesenchymal stem cell (MSC) biobanking. Both commercial and donor-derived bone marrow MSCs were assessed across master cell bank, working cell bank, and final product stages.

XT-Thrive consistently maintained higher cell viability during extended pre-freeze holding and post-thaw handling, with comparable or superior recovery relative to CS10. Preserved MSCs retained immunophenotypic identity, trilineage differentiation, genomic stability, and safety. Notably, enhanced osteogenic differentiation and elevated FGF2 and HGF secretion indicated improved preservation of therapeutic potential.

XT-Thrive represents a robust, scalable, and safer alternative to DMSO-based reagents, supporting xeno-free, clinically compliant MSC manufacturing for regenerative medicine.

## Data Availability

The original contributions presented in the study are included in the article/Supplementary Material, further inquiries can be directed to the corresponding author.
