# Supplementary material for: Evaluation of DMSO-free cryopreservation reagent XT-Thrive for establishment of mesenchymal stem cell bank platform
Source: Front Bioeng Biotechnol. 2026 Feb 5;14:1736526. doi: 10.3389/fbioe.2026.1736526 (PMC12916550; doi:10.3389/fbioe.2026.1736526)
Supplement: Supplementary file 1 [file Table1.docx]

Supplementary Table 1: Comparison of XT-Thrive with representative multicenter MSC cryopreservation approaches

| **Feature** | **Multicenter MSC Cryopreservation Studies (Mamo et al. 2024)** | **XT-Thrive (This Study)** |
| --- | --- | --- |
| 1. Cryoprotectant composition | DMSO-containing formulations | DMSO-free |
| 2. Primary evaluation metrics | Viability, immunophenotype, differentiation | Viability plus recovery, growth, functional performance |
| 3. Post-thaw attachment & recovery | Not systematically assessed | Directly assessed and improved |
| 4. Post-thaw proliferation | Limited evaluation | Enhanced recovery kinetics |
| 5. Functional potency indicators | Primarily identity-based assays | Secretome-associated and biological performance metrics |
| 6. Post-thaw processing | Requires washing and DMSO mitigation | No washing required |
| 7. Process robustness | Inter-site variability reported | Consistent functional outcomes |
| 8. Translational readiness | Established but constrained | Enhanced safety and scalability |
