# Supplementary material for: Evaluation of DMSO-free cryopreservation reagent XT-Thrive for establishment of mesenchymal stem cell bank platform
Source: Front Bioeng Biotechnol. 2026 Feb 5;14:1736526. doi: 10.3389/fbioe.2026.1736526 (PMC12916550; doi:10.3389/fbioe.2026.1736526)
Supplement: Supplementary file 2 [file Presentation1.pptx]

## Slide 1
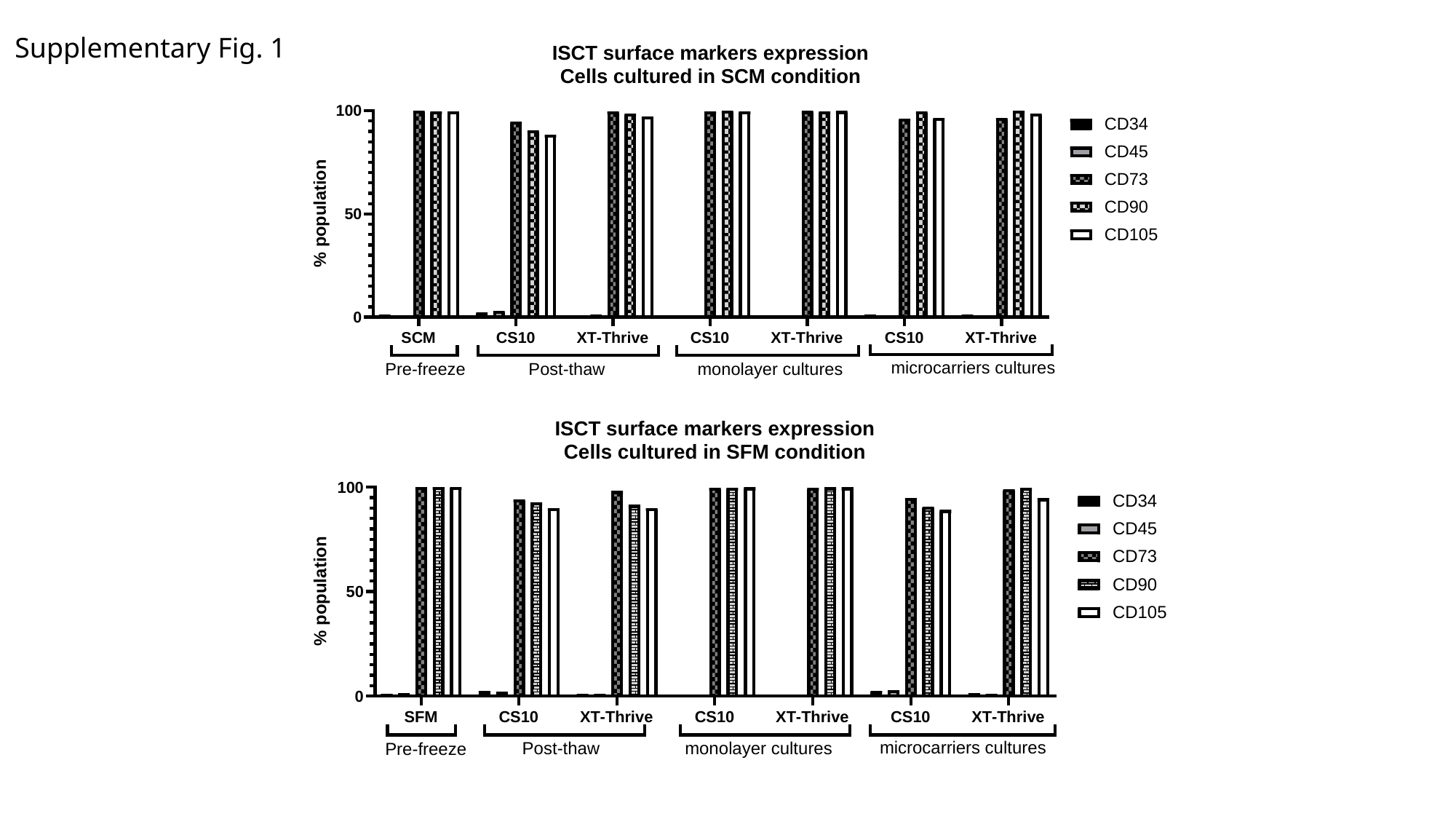

Supplementary Fig. 1

## Slide 2
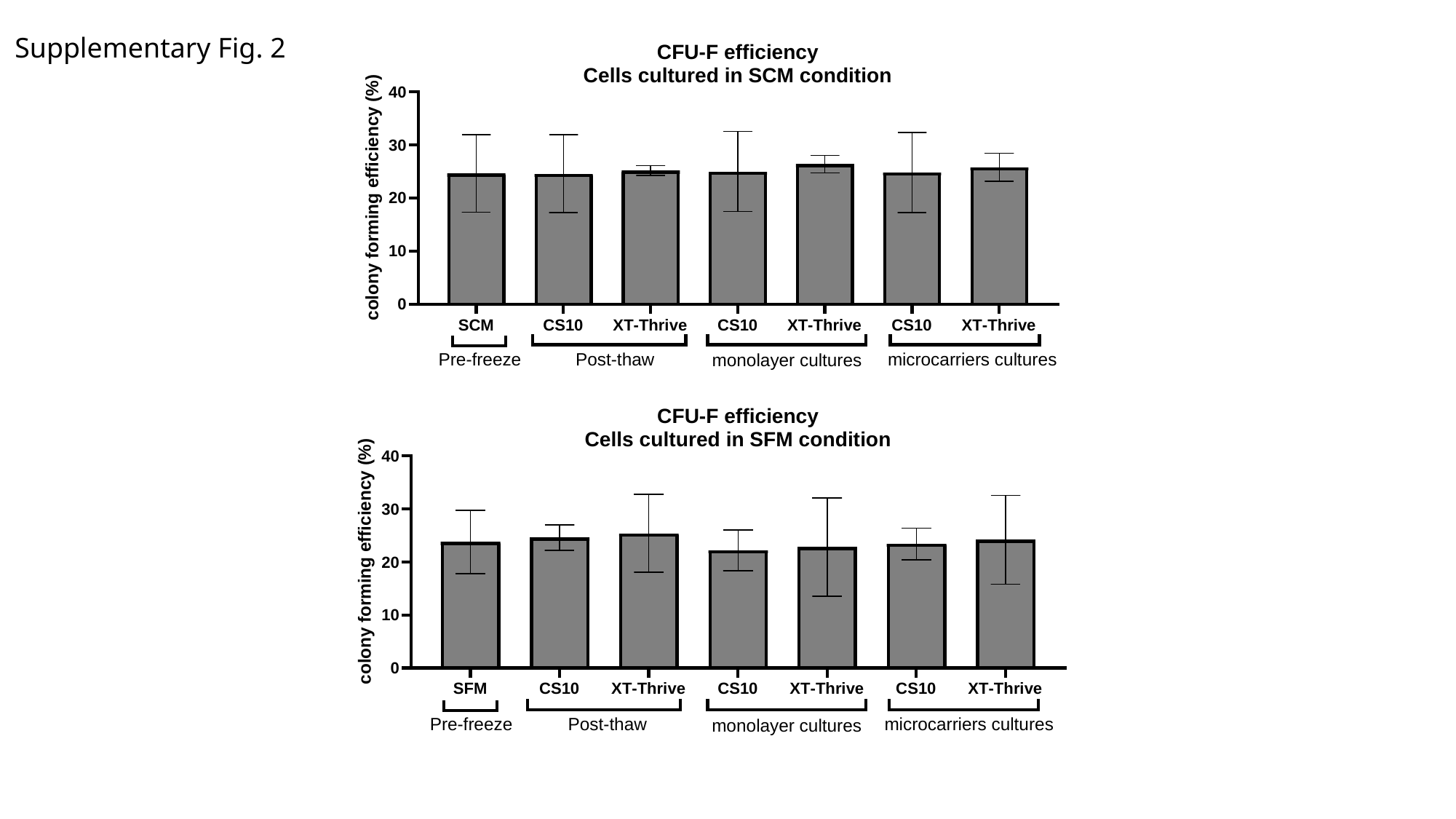

Supplementary Fig. 2

## Slide 3
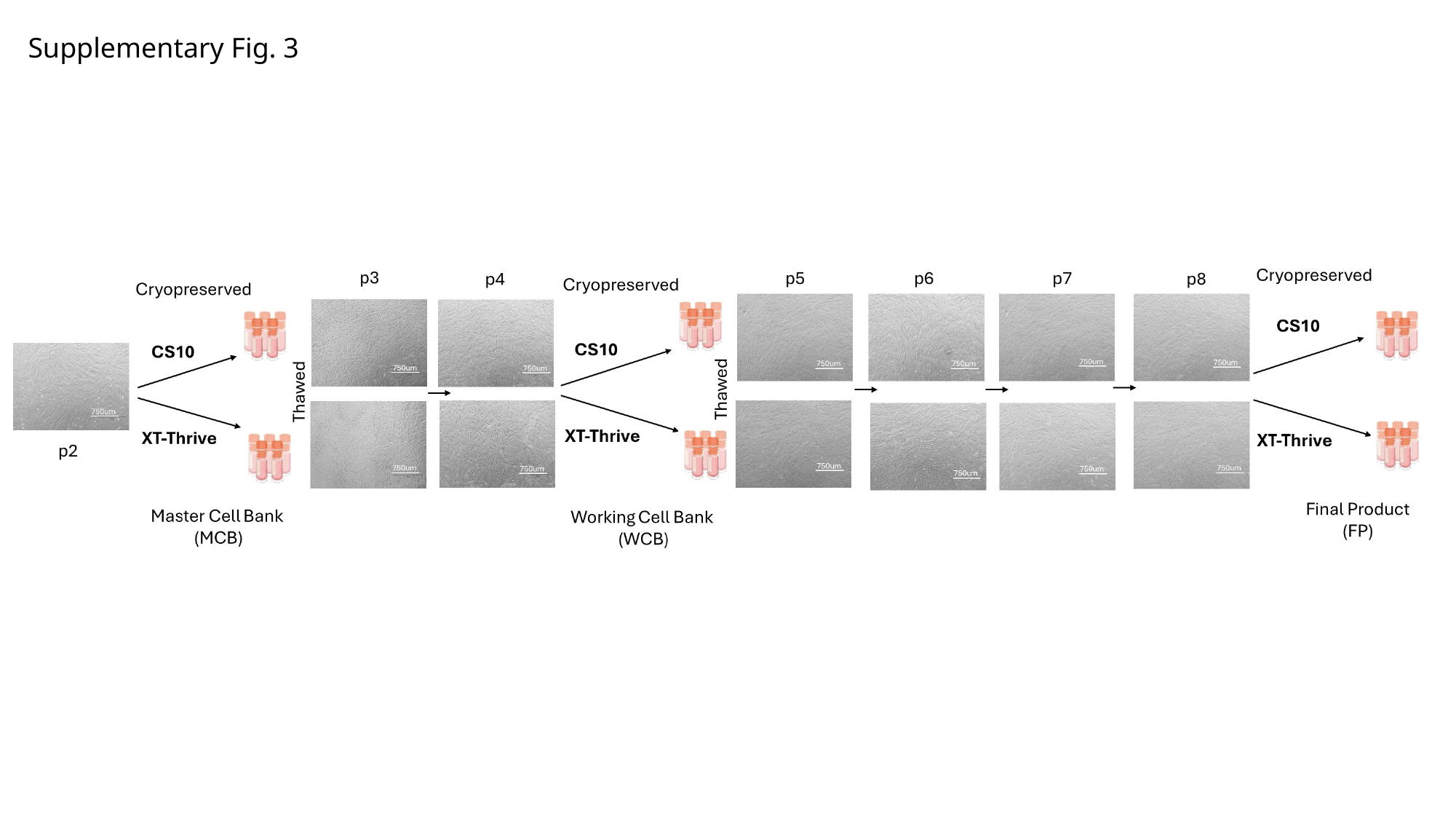

Supplementary Fig. 3

## Slide 4
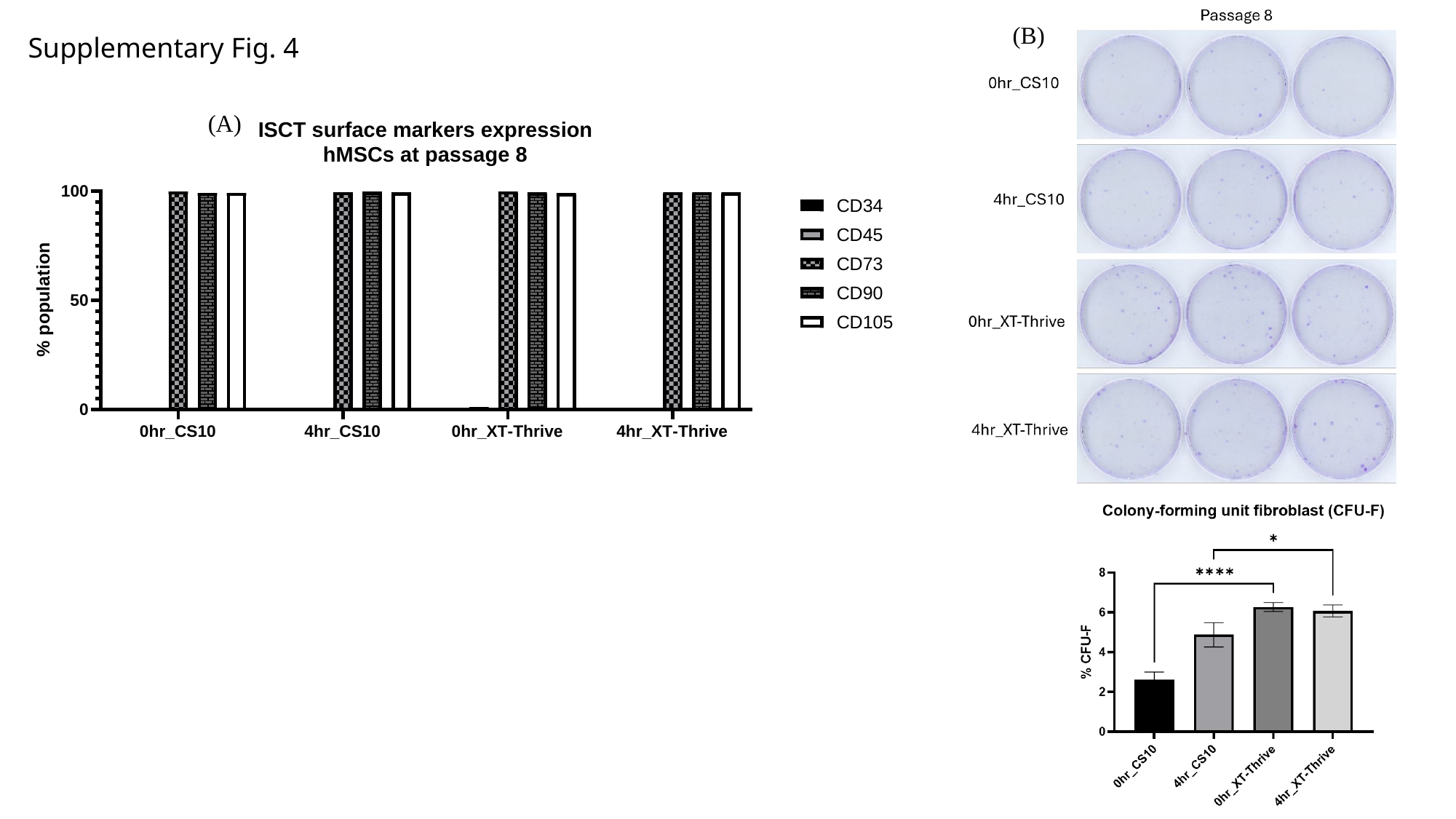

(B)
Supplementary Fig. 4
(A)

## Slide 5
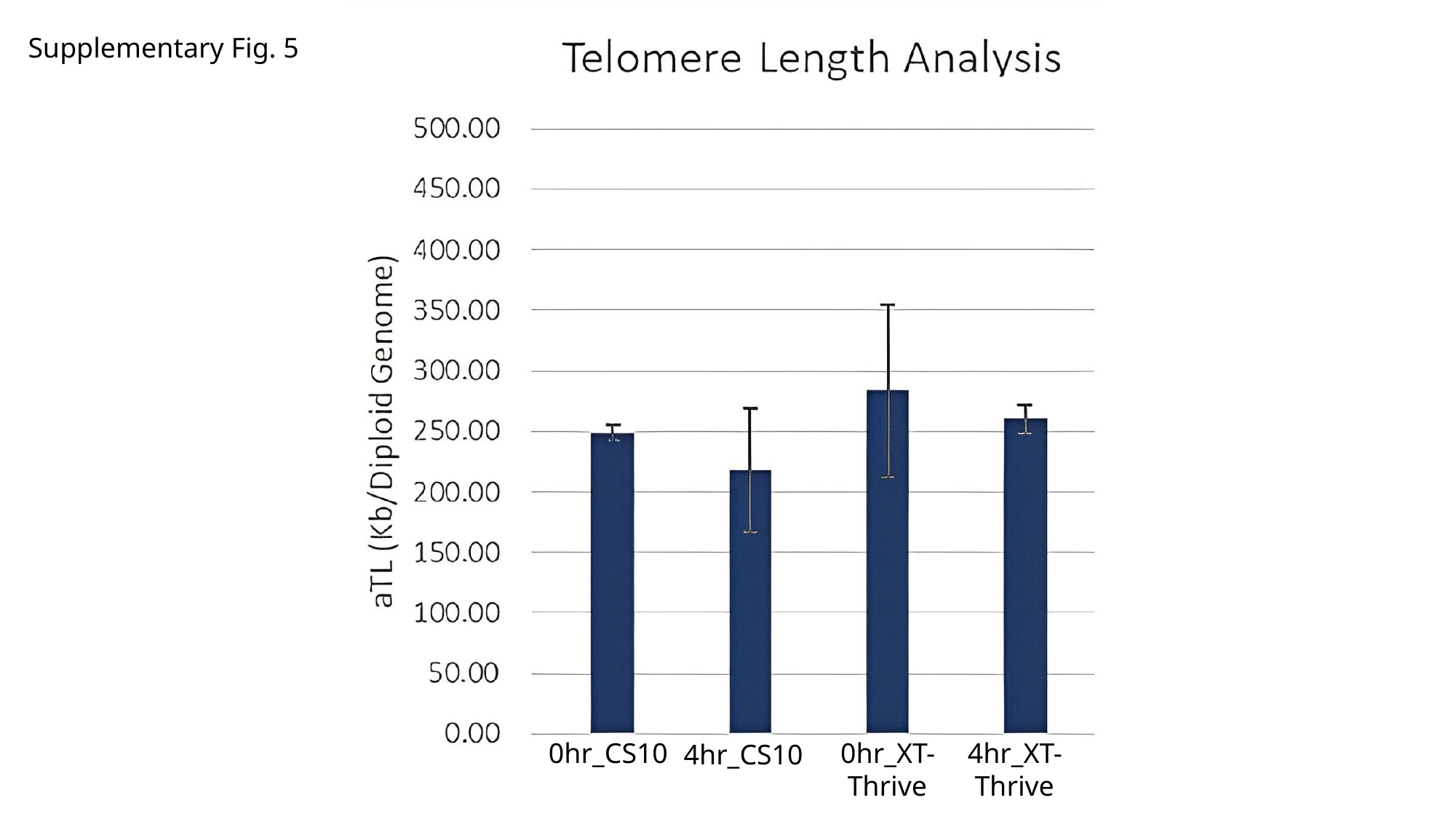

Supplementary Fig. 5
0hr_XT-Thrive
4hr_XT-Thrive
0hr_CS10
4hr_CS10

## Slide 6
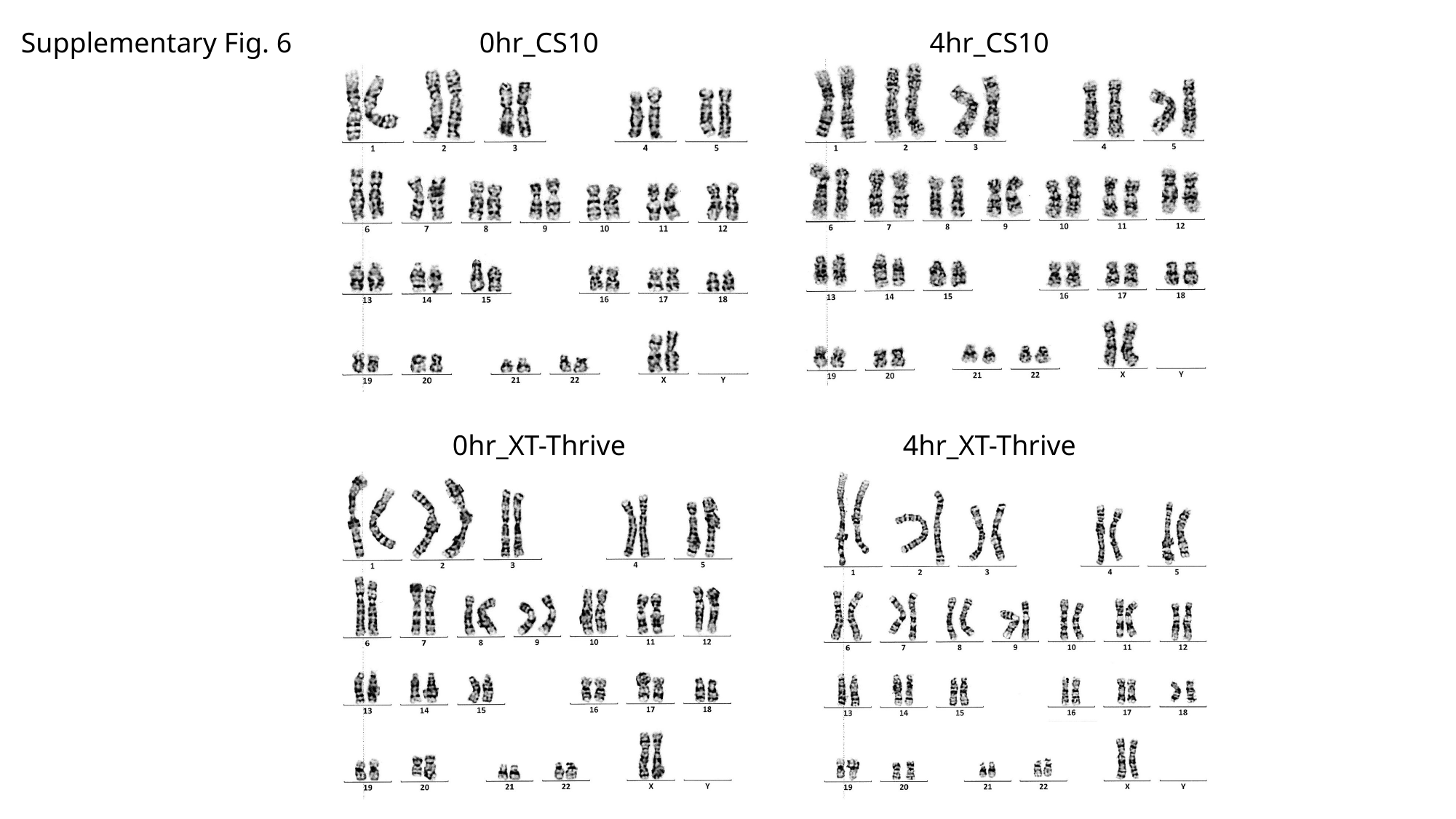

Supplementary Fig. 6
0hr_CS10
4hr_CS10
0hr_XT-Thrive
4hr_XT-Thrive

## Slide 7
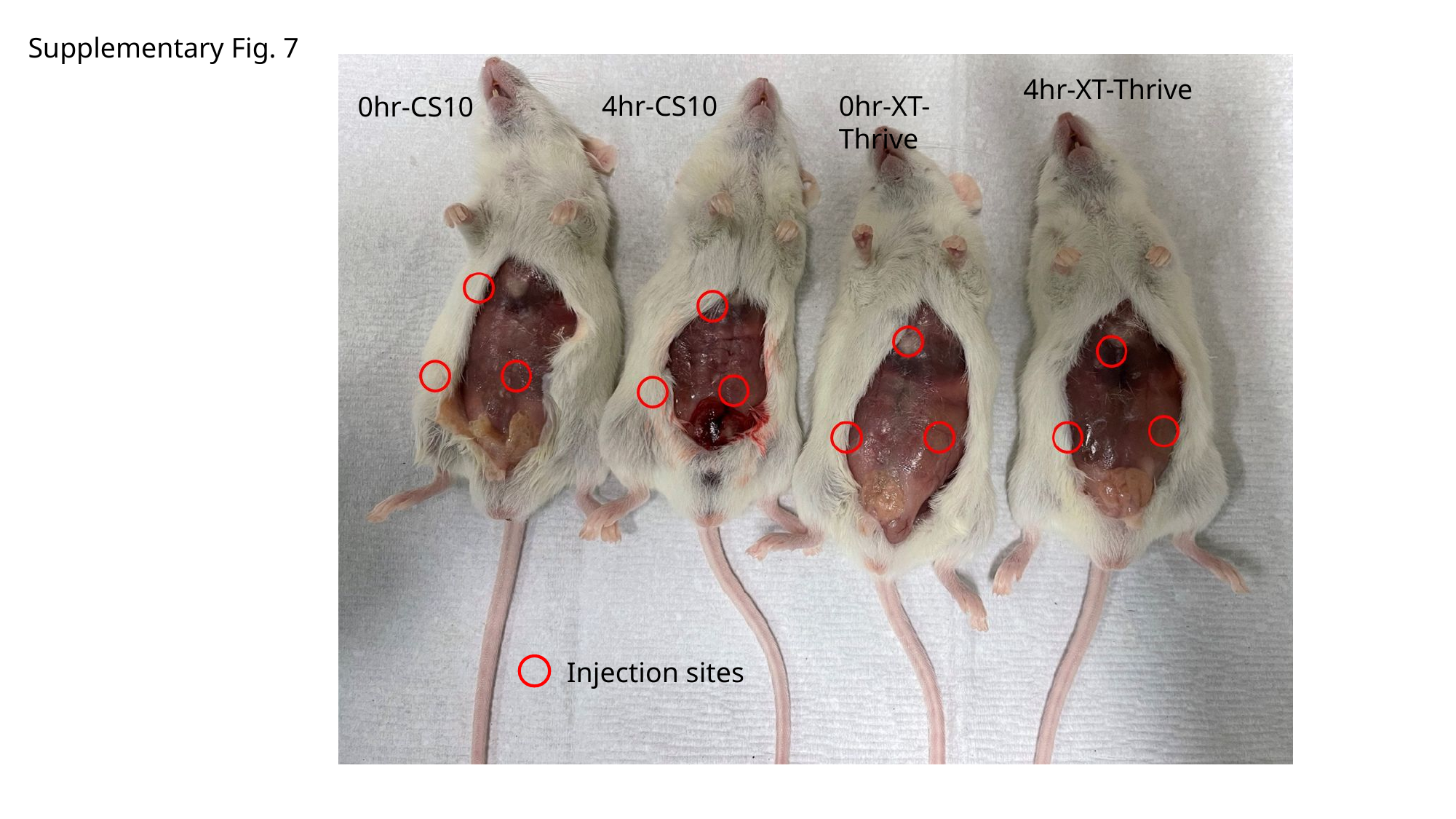

Supplementary Fig. 7
4hr-XT-Thrive
4hr-CS10
0hr-XT-Thrive
0hr-CS10
Injection sites
